# Enhanced transmission of malaria parasites to mosquitoes in a murine model of type 2 diabetes

**DOI:** 10.1186/s12936-016-1277-7

**Published:** 2016-04-21

**Authors:** Nazzy Pakpour, Kong Wai Cheung, Shirley Luckhart

**Affiliations:** Department of Medical Microbiology and Immunology, School of Medicine, University of California, Davis, CA USA; Department of Biological Sciences, California State University East Bay, 25800 Carlos Bee Blvd., Hayward, CA 94542 USA

**Keywords:** Type 2 diabetes, Malaria, *Plasmodium yoelii*, *Plasmodium berghei*, Mosquito, *Anopheles*

## Abstract

**Background:**

More than half of the world’s population is at risk of malaria and simultaneously, many malaria-endemic regions are facing dramatic increases in the prevalence of type 2 diabetes. Studies in murine malaria models have examined the impact of malaria infection on type 2 diabetes pathology, it remains unclear how this chronic metabolic disorder impacts the transmission of malaria. In this report, the ability type 2 diabetic rodents infected with malaria to transmit parasites to *Anopheles stephensi* mosquitoes is quantified.

**Methods:**

The infection prevalence and intensity of *An. stephensi* mosquitoes that fed upon control or type 2 diabetic C57BL/6 db/db mice infected with either lethal *Plasmodium berghei* NK65 or non-lethal *Plasmodium yoelii* 17XNL murine malaria strains were determined. Daily parasitaemias were also recorded.

**Results:**

A higher percentage of mosquitoes (87.5 vs 61.5 % for *P. yoelii* and 76.9 vs 50 % for *P. berghei*) became infected following blood feeding on *Plasmodium*-infected type 2 diabetic mice compared to mosquitoes that fed on infected control animals, despite no significant differences in circulating gametocyte levels.

**Conclusions:**

These results suggest that type 2 diabetic mice infected with malaria are more efficient at infecting mosquitoes, raising the question of whether a similar synergy exists in humans.

## Background

It is currently estimated that 22 million people are living with type 2 diabetes (T2D) on the African continent and this population is projected to double by 2030 [1]. Concurrently, there are over 250 million new malaria cases worldwide, the majority of which occur on the African continent. In contrast to developed countries, where the prevalence of T2D is greatest in individuals >60 years old, T2D in lesser developed countries is highly prevalent in individuals 40–60 years old [2], suggesting a durable epidemiological impact of T2D-associated increased malaria risk [3]. The rising prevalence of T2D in these regions can largely be ascribed to changes in lifestyle and urbanization, resulting in greater levels of obesity and physical inactivity [4, 5]. Published reports have shown that patients with T2D are 46 % more likely to be infected with *Plasmodium falciparum* [3]. Yet, the impact of T2D on malaria transmission remains unexamined.

Interestingly, *Plasmodium* infection improves glucose homeostasis in humans [6, 7], and in murine models of T2D it appears that glycosylphosphatidylinositols (GPIs) of the parasite are able to mimic the effects of endogenous insulin signalling [8–10]. Previous studies have examined the development of asexual stage *Plasmodium* in T2D mice and noted delayed development of parasitaemia [10, 11] and a correlation between obesity and increased resistance to cerebral malaria [11]. Therefore in a rodent model, T2D hosts with malaria may have altered duration and/or intensity of infection, possibly making them more efficient disease reservoirs. Although these studies clearly demonstrated that T2D and obesity can impact circulating levels of asexual parasites in mice, to date no published studies have examined the effects of T2D on malaria transmission to mosquitoes in any mammalian system. The evidence to suggest an effect of T2D on malaria transmission comes primarily from murine experiments, thus these experiments were conducted in similar models. This study provides the first evidence that a metabolic disorder can enhance transmission of malaria parasites to mosquitoes using a rodent model of T2D and malaria.

## Methods

### Mosquitoes

*Anopheles stephensi* (Indian strain) were reared and maintained at 27 °C and 80 % humidity. All mosquito rearing and feeding protocols were approved and in accordance with regulatory guidelines and standards set by the University of California Davis Institutional Animal Care and Use Committee. For transmission studies, 50 laboratory-reared, 3- to 5-day old, female mosquitoes were kept on water overnight and then allowed to feed for 15 min on anesthetized *Plasmodium*-infected animals prior to peak parasitaemia. Non-blood-fed mosquitoes were removed and after 10 days, midguts from mosquitoes were dissected into PBS and stained with 0.1 % mercurochrome for direct counting of *Plasmodium* oocysts.

### Mice

All animal experiments were approved by the UC Davis Institutional Animal Care and Use Committee and were performed in accordance with institutional guidelines on animal welfare. Six week-old female C57BL/6J and C57BL/6J db/db (B6.BKS(D)-*Lepr*^*db*^/J) mice were purchased from the Jackson Laboratories (Bar Harbor, ME, USA). Female CD-1 mice were purchased from Harlan Laboratories (Haslett, MI, USA). All mice were maintained in standard cages and received standard rodent chow (PMI lab chow number 5001) and sterile drinking water ad libitum.

### Parasites

*Plasmodium yoelii yoelii* 17XNL and *Plasmodium berghei* NK65 stocks were obtained from BEI Resources Malaria Research and Reference Reagent Resource (Manassas, VA, USA). Parasite stocks were made by passage in CD-1 mice. Mice were inoculated intraperitoneally with ~1 × 10^6^ infected red blood cells (RBCs) in 0.1 ml of saline. Daily percentages of parasitaemia in mice were calculated by counting the number of infected RBCs and dividing this number by the total number of RBCs in thin blood films stained with Giemsa (Sigma-Aldrich). Gametocytaemia was determined by visually quantifying gametocyte stage parasites and dividing this number by the total number of infected RBCs.

### Statistics

For mosquito transmission studies, data were analysed using Mann–Whitney test. Differences in prevalence of infection were determined by Chi square test. For parasitaemia and gametocytaemia, data were analysed by Student’s t test.

## Results

**Fig. 1:**
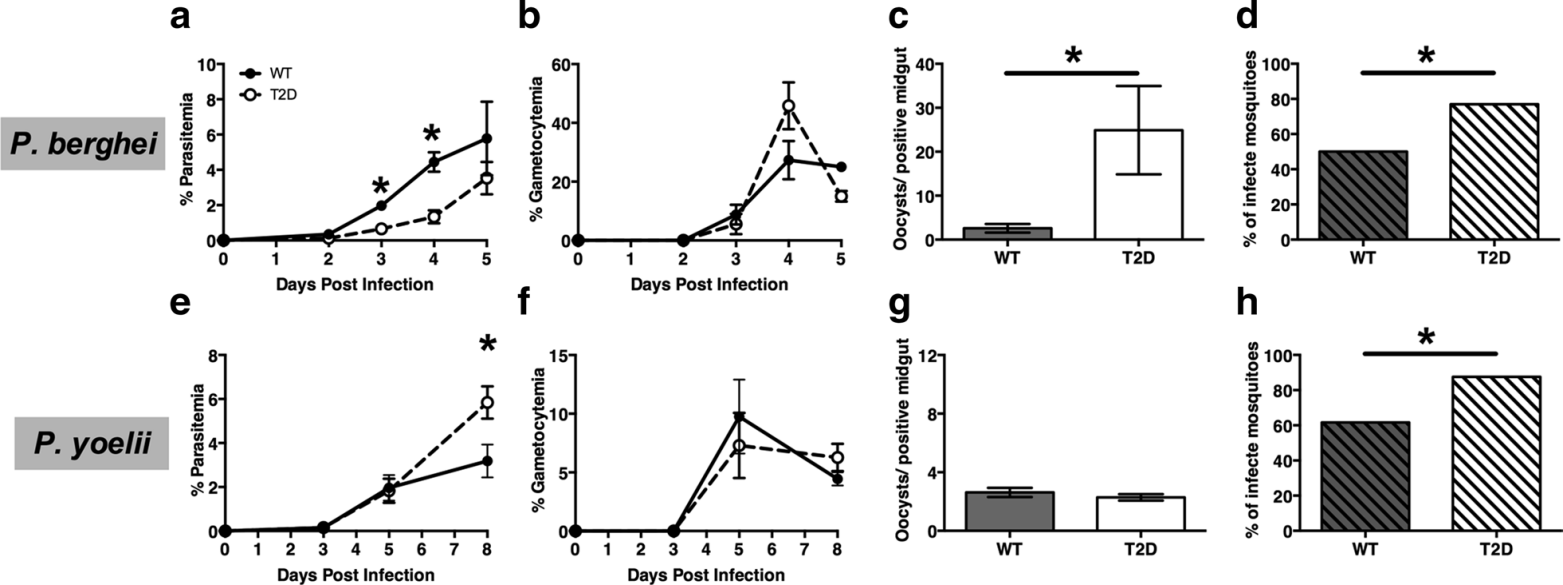
Parasitaemias, gametocytaemias and parasite transmission from wild-type (WT) and type 2 diabetic (T2D) C57BL/6 mice infected with malaria. **a**, **e** Parasitaemias and **b**, **f** gametocytaemias (mean ± SEM) were determined by visual counts from Giemsa-stained slides prepared from tail bleeds. n = 4–6, *p ≤ 0.05). **c**, **d**, **g**, **h** Prior to peak parasitaemia (day 5 for *Plasmodium berghei* and day 8 for *Plasmodium yoelii yoelii* 17XNL), mice were anesthetized and 50 mosquitoes/mouse were allowed to feed for 15 min. Mosquitoes were maintained on 10 % sucrose for 10 days at which point midguts were dissected and oocysts counted. n = 30–70 mosquitoes, *p ≤ 0.05

Similar to previously published reports, T2D mice infected with lethal *P. berghei* had decreased parasitaemias compared to infected wild-type controls, but gametocytaemias were no different from controls, except at day 4 post-infection (Fig. 1a, b). However, mosquitoes that fed upon *P. berghei*-infected T2D mice at day 5 post-infection had more than ten times the mean number of oocysts per positive midgut (Fig. 1c) compared to mosquitoes fed on infected wild-type mice. Further, nearly 60 % more mosquitoes were infected with at least one oocyst (Fig. 1d) after feeding on infected T2D mice compared to wild-type mice. In contrast, T2D mice infected with non-lethal *P. yoelii* parasites had significantly higher parasitaemias at day 8 compared to wild-type controls (Fig. 1e), but like *P. berghei*-infected mice, showed no significant differences in gametocytaemias (Fig. 1f). Interestingly, mosquitoes that fed upon *P. yoelii*-infected T2D and wild-type mice showed no differences in infection intensity (Fig. 1g). As with *P. berghei*, however, significantly more mosquitoes were infected with at least one oocyst following feeding on *P. yoelii*-infected T2D mice compared to infected wild-type mice (Fig. 1h). Thus, enhanced transmission was observed from T2D mice infected with lethal and non-lethal strains of malaria parasites.

## Discussion

These are the first data to show that T2D enhances the transmission of malaria parasites to a mosquito vector in a rodent model. In these studies, *P. berghei* NK65 were used as model for lethal cerebral malaria that more commonly affects children, and *P. yoelii* 17XNL, a model for non-lethal infection that is more typical of disease in adults. Increased transmission from T2D mice was observed, using different strains of murine malaria parasites with very different disease pathologies. Interestingly, no differences in infection intensity where observed in mosquitoes that fed upon *P. yoelii*-infected T2D and wild-type mice (Fig. 1g). This finding suggests that the factors that affect gametocyte development in the mosquito in a non-lethal *P. yoelii* infection are different than in a lethal *P. berghei* infection. Further studies are required to elucidate what these factors are and how they impact gametocyte development in the mosquito.

*Plasmodium* gametocytogenesis is defined by a succession of developmental stages, of which only mature male and female gametocytes are infectious to mosquitoes. Mature *P. yoelii* gametocytes sequester primarily in sub-dermal capillaries of the skin in mice, which provides protection from splenic clearance and promotes ingestion by mosquitoes during blood feeding [12]. Hosts with no detectable or sub-microscopic gametocytaemia retain the ability to infect mosquitoes, suggesting that significant numbers of mature gametocytes sequester in the skin [12–15]. As in data shown here, the association between circulating gametocyte densities in the mammalian host and infectivity to mosquitoes is not always linear [16]. Notably, altered parasite sequestration that is not correlated with peripheral gametocytaemia could contribute to enhanced transmission from T2D mice relative to wild-type mice. Therefore, future studies will examine if specific alterations in parasite sequestration occur in diabetic animals.

As the population of adults suffering from T2D continues to increase in malaria-endemic regions, these findings suggest that this co-morbidity could impact treatment and control efforts for both diseases in these regions. Indeed, health systems in low-resource countries have largely focused on acute rather than chronic care, which coupled with limited care access and delayed diagnosis of T2D contributes to more severe T2D disease and complications at the time of diagnosis [1]. Despite predictions that malaria-endemic African and Asian nations will face steeply rising incidences of T2D, few published reports have examined this co-morbidity. Indeed, beyond the activity of *Plasmodium* GPIs as insulin mimetics [8–10], little is known of how malaria might alter the pathology of T2D and how treatment of T2D might impact malaria infection and, more specifically, transmission. Thus, further investigations of T2D and malaria co-morbidity could inform future health interventions for these diseases.

## Conclusion

Type 2 diabetic mice infected with either *P. yoelii* or *P. berghei* resulted in a higher prevalence of infected *An. stephensi* when compared to wild-type controls. These mice also had significantly lower parasitaemias but no significant differences in circulating gametocyte levels. These data suggest that this co-morbidity in humans could significantly alter the infectious reservoir and transmission of malaria and should therefore be examined more closely.

## Abbreviations

GPIs: glycosylphosphatidylinositols
T2D: type 2 diabetes
*P. berghei*: *Plasmodium berghei*
*P. yoelii yoelii*: *Plasmodium yoelii yoelii*
RBC: red blood cells
